# Neural and Hormonal Control of Sexual Behavior

**DOI:** 10.1210/endocr/bqaa150

**Published:** 2020-08-26

**Authors:** Kimberly J Jennings, Luis de Lecea

**Affiliations:** Department of Psychiatry and Behavioral Sciences, Stanford University, Stanford, California

**Keywords:** reproductive behavior, gonadal hormones, sex hormones, activation, sex differences

## Abstract

Gonadal hormones contribute to the sexual differentiation of brain and behavior throughout the lifespan, from initial neural patterning to “activation” of adult circuits. Sexual behavior is an ideal system in which to investigate the mechanisms underlying hormonal activation of neural circuits. Sexual behavior is a hormonally regulated, innate social behavior found across species. Although both sexes seek out and engage in sexual behavior, the specific actions involved in mating are sexually dimorphic. Thus, the neural circuits mediating sexual motivation and behavior in males and females are overlapping yet distinct. Furthermore, sexual behavior is strongly dependent on circulating gonadal hormones in both sexes. There has been significant recent progress on elucidating how gonadal hormones modulate physiological properties within sexual behavior circuits with consequences for behavior. Therefore, in this mini-review we review the neural circuits of male and female sexual motivation and behavior, from initial sensory detection of pheromones to the extended amygdala and on to medial hypothalamic nuclei and reward systems. We also discuss how gonadal hormones impact the physiology and functioning of each node within these circuits. By better understanding the myriad of ways in which gonadal hormones impact sexual behavior circuits, we can gain a richer and more complete appreciation for the neural substrates of complex behavior.

Gonadal hormones play an essential role in the sexual differentiation of brain and behavior. Perinatal exposure to gonadal hormones guides neuronal growth, death, synaptogenesis, cytoarchitecture, chemoarchitecture, epigenetic modification, and many other brain characteristics to shape or “organize” sexually dimorphic neural circuits (1-6). Later exposure to gonadal hormones “activates” these circuits to promote expression of the relevant sex-typical behavior (5-7), and it is this deceptively simple concept we seek to spotlight in this mini-review. What does hormonal activation of a circuit mean at a mechanistic level? How is this implemented differently across circuit nodes and what is the consequence for behavior? We focus on sexual behavior as an ideal system in which to ask these questions: the behavior is ethologically relevant across species, easily studied in the laboratory, intensely dictated by hormonal status, and importantly—robustly expressed by both sexes. Therefore, herein we review key components of the neural circuitry underlying male and female sexual behaviors and highlight, whenever possible, recent advances in our understanding of the hormonal regulation of such circuits. We focus on literature from rodent models, due to their notable reliance on hormonal activation for sexual behavior and for the vast wealth of knowledge available from nearly a century of careful experimentation on these genetically tractable models.

## The Hormonal Regulation of Sexual Behavior

**Figure 1.**
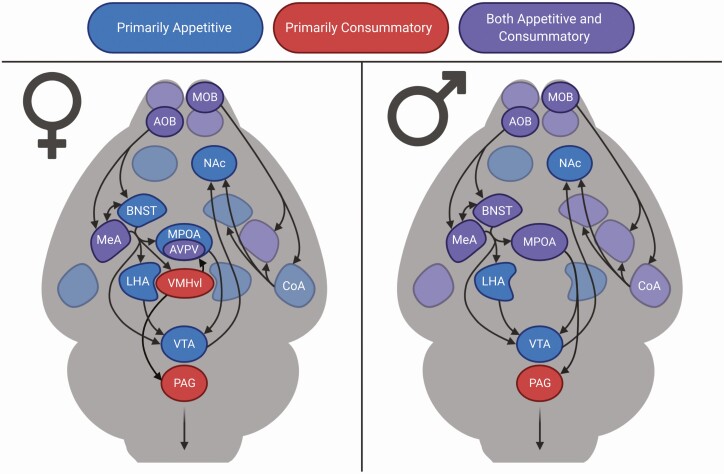
Neural circuits of male and female sexual behavior. Regions are color-coded based on major contributions to either appetitive or consummatory aspects of sexual behavior.

Sexual behaviors are often conceptualized into 2 categories: appetitive and consummatory (8). Appetitive sexual behavior entails actions that increase the likelihood of mating to occur and are thought to reflect sexual motivation. This includes approach, solicitation, or investigation of a potential mate and the exhibition of mate preference, or preference for an intact opposite-sex conspecific over a same-sex or gonadectomized conspecific. These behaviors can be displayed by both sexes with some species-specific differences (ie, specific solicitation behaviors may differ between sexes). On the other hand, consummatory sexual behavior entails the act of mating itself and is highly sexually dimorphic. In male rodents, this includes mounting and intromission, whereas in female rodents it is primarily adoption of the lordosis posture (stationary flexion of the spine and deflection of the tail permitting male intromission). Each of these aspects of sexual behavior is mediated by distinct but frequently overlapping neural substrates, which will be reviewed in the following sections (Fig. 1).

**Figure 2.**
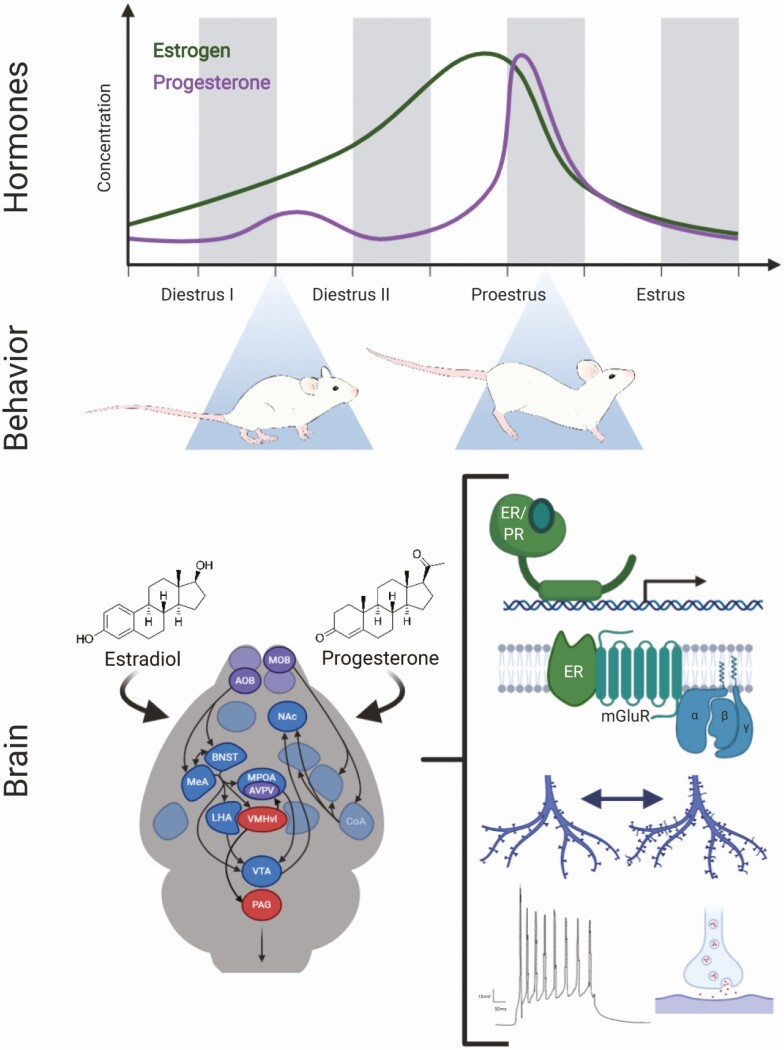
Hormonal control of female sexual behavior. The sequential rise in estrogen followed by progesterone across the estrous cycle (top) causes female rodents to be sexually receptive near ovulation (middle). This is mediated by an array of neurophysiological changes to the brain induced by hormonal signaling (bottom). Gonadal steroid hormones signal both through nuclear receptors and membrane-bound receptors (bottom right). This signaling regulates gene expression, structural remodeling, neuronal activity, and changes in synaptic properties in a region-specific manner.

Sexual behavior in both sexes is strongly regulated by circulating concentrations of gonadal steroid hormones, including androgens (testosterone), estrogens (estradiol), and progesterone. In rodents, this hormonal regulation is perhaps most obvious in females across the 4- to 5-day estrous cycle (Fig. 2). Estradiol concentrations are low during diestrus but build to a peak by the afternoon of proestrus, which, in conjunction with a daily circadian signal, triggers ovulation (9, 10). This is quickly followed by a sharp peak in progesterone produced by the corpus luteum, which declines by the following morning (11-13). The sequential rise in estrogen followed by progesterone primes the female brain and physiology for sexual motivation and behavior (“in heat” or in estrus) (14). Outside of this window, female mice, rats, and hamsters will not be receptive toward a male and will actively reject mating attempts. Consequently, by co-opting the neuroendocrine signals of ovulation to regulate sexual behavior, the female conserves energetic resources by only mating when maximally fertile. Importantly, this is distinct from old-world primates. For females of these species, including women, sexual behavior is expressed across the ovulatory cycle and the influence of gonadal hormones on sexual behavior is comparatively subtle (15-18).

Male sexual behavior is also dependent on sufficient basal circulating gonadal hormones, primarily testosterone and its metabolites. Many of the activational effects of testosterone on male sexual behavior in rodents can be attributed to its conversion into estradiol by the enzyme aromatase (19-22), which is highly expressed along sexual behavior circuits (23, 24). However, for the full and complete expression of male sexual behavior in laboratory models, both androgen and estrogen receptor signaling is required (25-28) (but see (26) for discussion of species differences). Typical laboratory models do not exhibit hormonal cycles that greatly impact male sexual behavior, but many other species do display seasonal cycles of reproductive activity, with commensurate changes to neuroendocrine, behavioral, and sensory systems (29-31). Furthermore, social experience (eg, social dominance, stress) can modify the hormonal milieu, even within laboratory models (32-34). Exposure to a potential mate or to a social challenge elicits an acute and transient increase in testosterone above basal levels in males across species (35-38). Such socially induced testosterone pulses have been hypothesized to modify future behavior in several ways, including by promoting territory formation, promoting future winning (ie, the winner effect), modifying social vigilance, reducing anxiety, and potentially facilitating responses to the social situation through rapid, nongenomic actions (38-41). Thus, adults of both sexes can experience fluctuations in gonadal hormones that may impact brain and behavior.

## The Main and Accessory Olfactory Systems

Animals rely on pheromone signaling to communicate social information essential for reproductive behavior. These chemosignals are detected by the complementary but distinct main and accessory olfactory systems (MOS, AOS) (42, 43). Within the MOS, sensory neurons in the main olfactory epithelium (MOE) detect volatile odorants and relay this information to the main olfactory bulb. Accordingly, the MOS is thought to be particularly important for initial approach behavior and inherent social attraction based on volatile cues (44, 45). On the other hand, within the AOS, sensory neurons of the vomeronasal organ (VNO) detect pheromones transmitted through close contact with a conspecific. This information is then conveyed to the accessory olfactory bulb, which sends projections to the extended amygdala that are considered particularly important for pheromonal elicitation of reproductive behavior and neuroendocrine responses (42, 46). Although these 2 systems are anatomically distinct and respond to different classes of pheromones, information from the MOS also reaches the extended amygdala through the cortical amygdala and a minor but direct projection from the main olfactory bulb (44, 47).

Both the MOS and AOS are essential for the complete and appropriate display of sexual behavior. Male and female pheromones elicit distinct sex-specific patterns of activation within both systems (47-50). Lesions or genetic disruption of either the MOE or VNO disrupt sociosexual behavior in both sexes (51-56). For example, genetic mutation of the ion channel TrpC2 abolishes pheromone signal transduction in the VNO. TrpC2^-/-^ mice inappropriately mount same- and opposite-sex conspecifics at high levels (57-59), despite TrpC2^-/-^ mice, or even animals with complete VNO lesions, retaining the ability to discriminate male versus female odorants through the MOS (54, 60-62). Thus, the AOS is considered particularly important for regulating the expression of specific social behaviors toward the appropriate target (eg, to mate or to attack) (46).

Recent work has shed light on how fluctuations in sex hormones across the estrous cycle shape sensory processing in the AOS. Estradiol regulates expression of ion channels within the VNO and rapidly modifies vomeronasal sensory neuron (VSN) responses to pheromones (63-65). Furthermore, Dey et al reported that moderate concentrations of progesterone (approximately that of diestrus, ~13 ng/mL) act to silence VSNs (66). Intriguingly, progesterone-mediated silencing was seen in VSNs sensitive to male pheromones but not VSNs that were sensitive to predator odor, revealing hormonal modulation specifically of socially relevant sensory input. However, this study did not test the effect of progesterone at high concentrations seen during late proestrus (~50 ng/mL (67-69)), so whether the peri-ovulatory progesterone surge might counterintuitively inhibit pheromone-sensing VSNs or whether this effect is dose-dependent remains to be tested. Regardless, changing concentrations of circulating estrogen and progesterone across the estrous cycle can clearly modulate the female’s earliest sensory detection of male cues. In males, testosterone has been shown to increase activation of both the AOS and MOS in response to female pheromones (70, 71), although there is little data available on the underlying molecular mechanisms mediating this effect (63).

## The Extended Amygdala

The medial amygdala (MeA) is a major target of the AOS and minor target of the MOS that has been strongly implicated in mediating sexual behavior (47, 72). In particular, the posterodorsal subdivision of the MeA (MeApd) expresses a high density of sex hormone receptors and is well accepted to be activated during mating or by exposure to opposite-sex pheromones (51, 72-75). Indeed, recent work has demonstrated that neurons of the MeA differentially encode male versus female cues (76, 77), and that this separable encoding is shaped by experience (78). The MeApd appears to regulate aspects of both mate preference and consummatory sexual behavior. Lesions of the MeApd disrupt mate preference in both sexes (79-82). MeApd lesions also disrupt sexual behavior in males (83-86). In females, MeA lesion or MeA chemo-inhibition reduces but does not eliminate lordosis behavior (82, 87), and MeA lesions do not impact the amount of mounts or intromissions received in a mating assay (88). These data indicate that the MeA promotes lordosis responses but is not essential for its expression.

Recent studies targeting genetically identified MeApd subpopulations have highlighted the complex role of the MeApd in regulating multiple social behaviors. Disrupting oxytocin signaling in aromatase-expressing MeApd neurons eliminated mate preference in males (77). However, ablating these neurons did not impair sexual behavior in either sex, although it did impair aggressive behavior (89). GABAergic MeApd neurons can promote mounting or aggression depending on stimulation intensity (90). On the other hand, optogenetic inhibition of MeApd GABAergic neurons did not interrupt intromission, suggesting that while these neurons may facilitate mount initiation, they are not necessary for continued sexual behavior. Finally, chemogenetic stimulation of kisspeptin-expressing MeApd neurons in males promoted social investigation without impacting consummatory sexual behavior (91). Thus, it seems likely that there exists multiple parallel or combinatorial subcircuits involving the MeApd that guide expression of the appropriate social behavior to a given stimulus.

Another component of the “extended amygdala,” the bed nuclei of stria terminalis (BNST), is also implicated in the control of sexual behavior. The BNST receives direct and indirect input from the AOS, exhibits a high density of steroid hormone receptors, and contains numerous overlapping neuropeptide subpopulations (72, 92-95). The BNST contains spatially and genetically segregated neuronal subpopulations capable of driving either aversive or appetitive behavioral states. Of note, male cholecystokinin (CCK)-expressing medial BNST neurons are both preferentially activated by opposite-sex odorants and produce reinforcement when stimulated (93), which could conceivably contribute to the expression of mate preference. Lesions to the BNST largely delay or slow mating in males (96-102), with seemingly greater effects in naïve animals (101, 102). Indeed, aromatase-expressing neurons in the principal component of the BNST (BNSTpr^Aro^) were recently reported to exhibit distinct activity patterns in response to male versus female conspecifics in naïve males (103), distinguishing it from the MeA, which requires social experience to encode sex discrimination (78). Inhibiting or ablating these neurons eliminated mate preference and reduced consummatory sexual behavior and aggression, whereas stimulating these neurons (in line with the endogenous response to females) promoted mounting directed to male conspecifics (103). Thus, the authors propose that BNSTpr^Aro^ neurons represent a neural substrate of sex recognition, vital information for the selection of appropriate social responses. Interestingly, this role seems unique to males, as BNSTpr^Aro^ neurons in females do not show similar activity patterns or effects on behavior (103).

Gonadal hormones regulate several aspects of neuronal physiology within the extended amygdala. First, local replacement of testosterone or estradiol to the MeA in gonadectomized males facilitates expression of sexual behavior (104-108), indicating that hormonal signaling within this region promotes activation of sexual behavior circuits. Second, adult gonadal hormones support sexual dimorphism in regional volume and soma size within the MeA (109, 110). Third, fluctuations in estrogen and progesterone across the estrous cycle modulate synaptic and electrophysiological features of neurons within these regions (111-114). For example, estrogen has been reported to selectively regulate the excitability of afferents to the MeA in a source-specific manner (115). Similarly, the excitatory:inhibitory balance of inputs onto the MeApd varies across the estrous cycle (116). These changes could regulate the computational weight of various MeA inputs across the estrous cycle and bias sexual interest to the estrus period. Finally, gonadal hormones regulate expression of various neuropeptides within the MeA and BNST of both sexes (117-122), which likely further impacts neuromodulatory control of information processing. This includes expression of the neuropeptide CCK within the BNST (118), indicating that the reward-promoting BNST^cck^ population discussed above (93) is likely regulated to some degree by gonadal hormones. However, how these hormone-driven changes in neuronal physiology relate to observable changes in sociosexual behavior remain unclear.

## Medial Hypothalamic Centers

Looking downstream of the extended amygdala, the medial preoptic area (MPOA) is essential for the display of male sexual behavior. This has been demonstrated by decades of lesion, stimulation, and pharmacological studies which have been excellently reviewed in detail elsewhere (28, 123). The MPOA receives a wide array of afferents and is thought to integrate information from both olfactory systems, hormonal state via rich expression of steroid receptors, and sensory input from the genitals (123). The MPOA comprises a heterogenous mix of cell types and exhibits distinct input/output patterns across different subregions (124-129), complicating functional dissection of this region. A recent study used fiber photometry to demonstrate *esr1* (the gene for estrogen receptor α)-expressing MPOA neurons (MPOA^esr1^) are active during social investigation and that activity increased further during mounting. Optogenetic manipulation of MPOA^esr1^ neuronal activity bi-directionally regulated expression of mounting behavior (130). MPOA^esr1^ neurons also regulated expression of maternal behavior in both sexes, indicating that *esr1* expression marks a broader MPOA population containing substrates of multiple hormonally regulated social behaviors. Interestingly, manipulating MPOA^esr1^ neuronal activity in males did not impact time spent investigating a female during mating assays, suggesting basic social interest is unaffected. Indeed, the MPOA’s role in sexual motivation was formerly controversial (131), as several studies reported that MPOA lesions did not impair male interest in female conspecifics (100, 132, 133). However, additional MPOA lesions studies across species have reported deficits in mate preference and pursuit behavior (134-139). Thus, it is now believed that the MPOA facilitates sexual motivation in addition to mediating consummatory sexual behavior in males.

On the other hand, the MPOA likely does not play a strong role in modulating lordosis behavior in females. Lesions of this region typically do not impair and may even promote expression of lordosis (81, 140-143). However, the MPOA does support sexual motivation in females, as MPOA lesions disrupt approach behavior and mate preference (81, 88, 143-147). Indeed, McHenry et al recently characterized a MPOA→ ventral tegmental area (VTA) circuit that promotes social interest in both sexes and is regulated by ovarian hormones across the estrous cycle (148). Female neurotensin-expressing MPOA neurons (MPOA^nts^) respond preferentially to male odors, and estradiol exposure enhanced both MPOA^nts^ intrinsic excitability and MPOA^nts^ responsiveness to male cues. Stimulation of either MPOA^nts^ neurons or MPOA^nts^→VTA fibers was reinforcing in both sexes. Estradiol exposure (whether at proestrus or with exogenous treatment) enhanced this effect in females. Finally, bi-directionally manipulating MPOA^nts^ activity regulated time spent investigating an opposite-sex conspecific and the expression of mate preference (148). These data support the role of the MPOA in appetitive sexual behavior in both sexes and provide a mechanism by which changes in ovarian hormones across the estrous cycle can gate female sexual motivation. Complementarily, estrogen can also act to inhibit female sexual behavior outside of the appropriate proestrus period. Briefly, estrogen acts in the arcuate nucleus via membrane-bound signaling to drive release of β-endorphin in the medial preoptic nucleus (MPN), which is embedded within the MPOA. This activation of µ opioid receptors in the MPN inhibits female sexual behavior. At proestrus, progesterone acts to de-activate MPN µ opioid receptors and thereby facilitate the transition to sexual receptivity (reviewed in (149, 150)). Thus, estrogen can both augment mate preference during proestrus and inhibit receptivity in the absence of the proestrus progesterone peak.

The ventrolateral subdivision of the ventromedial hypothalamus (VMHvl) is essential for female lordosis behavior. Decades of work by Pfaff and colleagues characterized a lordosis reflex circuit with the VMH as a necessary and sufficient hormone-sensitive locus, which signals to midbrain premotor regions such as the periaqueductal gray (reviewed in (14, 149, 154, 155)). Local infusion of estradiol and progesterone into the VMHvl stimulates female sexual behavior (151-153). With advances in genetic access to specific cell types, progesterone receptor-expressing neurons in the VMHvl (VMHvl^PR^) have emerged as particularly essential for female sexual behavior (156). Recently, Inoue et al described a VMHvl^PR^→anteroventral periventricular nucleus (AVPV) circuit which is necessary for female receptivity and exhibits structural remodeling across the estrous cycle (157). Female VMHvl^PR^ neurons are preferentially active during mating and when investigating males. Indeed, inhibiting VMHvl^PR^ neurons reduced receptivity in hormonally primed females, but, surprisingly, stimulating VMHvl^PR^ neurons failed to promote lordosis behavior in unprimed mice. Further investigation revealed that both exogenous estrogen exposure and the natural increase in estrogen during the estrous cycle increases the number of presynaptic terminals in the AVPV from VMHvl^PR^, with striking consequences for the functional connectivity between the VMHvl and AVPV. Inhibition of this pathway reduces lordosis behavior, indicating that the estrogenic gating of the VMHvl^PR^→AVPV circuit likely has behavioral consequences for limiting receptivity to behavioral estrus. Consistent with these data, the partially overlapping VMHvl^esr1^ neuronal population has been reported to contain mating- and fighting-activated subpopulations, with the mating-related but not fighting-related VMHvl^esr1^ subpopulation projecting strongly to the AVPV (158). Within the AVPV, there is further cell-type specificity: ablating AVPV tyrosine hydroxylase neurons did not impair female sexual behavior (159), whereas ablating AVPV kisspeptin neurons eliminated both mate preference and lordosis (160). AVPV kisspeptin neurons are themselves strongly regulated by ovarian hormones and play a prominent role in the neuroendocrine cascade regulating ovulation (161).

## Reward Systems

It will surprise no one that sexual activity is rewarding and reinforcing. Activity of the mesolimbic dopamine system, namely dopamine release by the VTA into the nucleus accumbens (NAc), is thought to signal motivational salience of a stimulus, encode reward predictions, and facilitate reinforcement learning (162). The VTA receives significant input from the MPOA, MeA, and BNST in both sexes (163). Numerous groups have reported elevated dopamine release in the NAc upon presentation of a potential mate and during active mating in both males and females (164-172). With recent methodological advances enabling greater temporal resolution, we see escalating amount of dopamine release in a male as the mating behavioral suite progresses, with the highest release associated with ejaculation (173, 174). In female rats, dopamine release is dependent on the testing environment. If allowed space to retreat, female rats will pace the mating interaction such that she receives intromissions at an interval which will maximize her reproductive success (175-178). Accordingly, sexual reward and elevated dopamine release during mating is most robustly observed when mating proceeds at the female’s preferred pace, regardless of her display of lordosis behavior under nonpaced conditions (179-183). This is consistent with VTA dopamine release reflecting motivational state and sexual reward separate from motor actions.

VTA→NAc signaling likely supports expression of social interest and mate preference. Manipulating VTA→NAc neuronal activity bi-directionally modulates time spent investigating a conspecific (184). Furthermore, lesioning dopaminergic inputs to the NAc or blocking D1 receptor signaling in the NAc eliminated mate preference (185, 186). Conversely, stimulating dopamine release within the NAc rescued mate preference in TrpC2^-/-^ mice, which have impaired pheromone detection (185). Another region of the ventral striatum, the medial olfactory tubercle, has been implicated in natural reinforcement and in encoding the innate hedonic valence of odorants (187, 188). Accordingly, lesions that include the medial olfactory tubercle or chemogenetic inhibition of the medial olfactory tubercle impair female mate preference for male odorants (189, 190).

Despite limited expression of steroid receptors in the VTA and NAc, estrogen is generally accepted to augment dopaminergic signaling in this pathway (191, 192). VTA dopaminergic neuron basal firing rate is enhanced and more dopamine released into the NAc during behavioral estrus (193-195). Several electrophysiological properties of NAc medium spiny neurons have been reported to either vary across the estrous cycle or to be rapidly modulated by estradiol exposure (192, 196, 197). Furthermore, like in many of the regions discussed above, estrogen exposure also regulates spine density in the NAc (198, 199). These effects seem to be region-specific within the striatum, as estrogenic effects on spine density and excitatory synaptic properties observed in the NAc were not seen in the caudate-putamen (197, 198). The effects of gonadal hormones on NAc physiology is not limited to females, as long-term treatment with androgens in males also modulates NAc dendritic spine density (200).

Another intriguing region that projects heavily to the VTA is the lateral hypothalamic area (LHA). This highly heterogeneous region modulates motivational drives relevant to many behaviors (201-203). The LHA has been implicated in both promoting and inhibiting sexual behavior in males. Serotonin is released into the LHA after ejaculation in males, and pharmacological elevation of serotonin within the LHA both attenuates dopamine release in the NAc in response to a female and inhibits sexual behavior (204, 205). Thus, serotonin signaling in the LHA may contribute to the postejaculatory refractory period, during which male sexual motivation is tightly suppressed. On the other hand, LHA neurons that express the neuropeptide hypocretin (hcrt, also known as orexin) promote goal-directed action in response to a wide array of stimuli (206-208). LHA^hcrt^ neurons have been suggested to promote male sexual behavior based on increased activity during mating and pharmacological manipulation of hcrt signaling (209, 210). Interestingly, hcrt expression is also hormonally regulated in both sexes and varies across the estrous cycle (210-212), suggesting another avenue by which gonadal steroids can orchestrate sexual motivation.

## Conclusion

Although for ease of explanation we have presented the above discussion as a forward flow of information from olfactory systems to the extended amygdala to the medial hypothalamus and reward systems, reality is not so straightforward. All the regions discussed above, and several others, send projections to each other, allowing for the possibility of feedback and crosstalk amongst systems. Furthermore, most of these regions have been implicated in the control of multiple social behaviors beyond sexual behavior, including territorial aggression, parental behavior, or maternal aggression. Indeed, based on neuroanatomical interconnections, strong steroid hormone receptor expression, and overlapping patterns of activation across social behaviors, the existence of a “social behavior network” was proposed (72). This network is highly conserved across taxa, providing a useful framework for comparative analysis (213, 214). This perspective has also proven useful for conceptualizing hormonal regulation of social behavior circuits. Through this lens, gonadal hormones act to tune connections and activity patterns across the social behavior network and thus shift the likelihood of a particular social response (72, 215). Indeed, as discussed above, gonadal hormones regulate a myriad of structural, electrophysiological, and genetic elements which converge to augment or attenuate circuit activity and behavioral output (Fig. 2). Recent and continued development of increasingly powerful tools is enabling unprecedented dissection of neuronal subcircuits with genetic precision. With this enhanced understanding of the neural circuits of behavior, we have a stronger foundation from which to probe the hormonal regulation of complex behavior.

## Data Availability

Data sharing is not applicable to this article as no datasets were generated or analyzed during the current study.

## Abbreviations

AOS: accessory olfactory system
AVPV: anteroventral periventricular nucleus
BNST: bed nuclei of stria terminalis
CCK: cholecystokinin
hcrt: hypocretin
LHA: lateral hypothalamic area
MeA: medial amygdala
MeApd: posterodorsal subdivision of the medial amygdala
MOE: main olfactory epithelium
MOS: main olfactory system
MPN: medial preoptic nucleus
MPOA: medial preoptic area
NAc: nucleus accumbens
VMHvl: ventrolateral subdivision of the ventromedial hypothalamus
VNO: vomeronasal organ
VSN: vomeronasal sensory neuron
VTA: ventral tegmental area

## Additional Information

***Disclosure Summary:*** The authors report no conflict of interest.
